# Cinnamaldehyde Inhibits Leptin-Induced MMP-1 by Modulating Leptin Receptor/STAT3 and Blocking RhoA/NF-κB Pathways in Human Intervertebral Disc Stem Cells

**DOI:** 10.3390/ijms26199819

**Published:** 2025-10-09

**Authors:** Kuo-Feng Hua, Hsin-Chiao Yu, Hsien-Ta Hsu

**Affiliations:** 1 Department of Biotechnology and Animal Science, National Ilan University, Ilan 260007, Taiwan; kfhua@niu.edu.tw; 2 Department of Medical Research, China Medical University Hospital, China Medical University, Taichung 404328, Taiwan; 3 Division of Neurosurgery, Taipei Tzu Chi Hospital, Buddhist Tzu Chi Medical Foundation, New Taipei City 231, Taiwan; tch34274@tzuchi.com.tw; 4 School of Medicine, Buddhist Tzu Chi University, Hualien 970, Taiwan

**Keywords:** intervertebral disc degeneration, leptin, matrix metalloproteinase, intervertebral disc cartilage endplate-derived stem cells, cinnamaldehyde

## Abstract

Obesity is a recognized risk factor for intervertebral disc (IVD) degeneration, a condition characterized by the progressive loss of extracellular matrix components in the nucleus pulposus. Elevated circulating leptin levels in obese individuals contribute to this degeneration by upregulating matrix metalloproteinase-1 (MMP-1) expression. Targeting MMP-1 expression with low-toxicity natural compounds may provide a promising strategy to prevent or mitigate IVD degeneration. In this study, we examined the effects of cinnamaldehyde (CA), a natural compound derived from *Cinnamomum osmophloeum* Kaneh, on leptin-induced MMP-1 expression in human IVD cartilage endplate-derived stem cells (SV40 cell line). Our results showed that leptin induced MMP-1 expression via activation of leptin receptor-mediated JAK2/STAT3, JAK2/RhoA/STAT3, and RhoA/ERK1/2/NF-κB signaling pathways. CA significantly reduced MMP-1 expression by inhibiting phosphorylation of the leptin receptor and STAT3 and blocking RhoA and NF-κB activation, without affecting JAK2 and ERK1/2 phosphorylation. These findings suggest that CA suppresses leptin-induced MMP-1 expression by modulating specific signaling pathways, highlighting its potential as a therapeutic agent for IVD degeneration associated with obesity.

## 1. Introduction

Intervertebral disc (IVD) degeneration is a prevalent age-related condition characterized by the progressive loss of collagens and proteoglycans in the nucleus pulposus. The decline in proteoglycan content compromises the disc’s capacity to resist mechanical loading, leading to structural weakening and functional impairment [1]. Clinically, IVD degeneration most commonly affects the cervical and lumbar regions in older adults and is a major contributor to lower back pain, often progressing to severe mobility limitations [2]. While the underlying pathogenesis remains incompletely understood, aging and obesity are recognized as major risk factors [3,4,5].

Obesity, frequently accompanied by metabolic disorders such as type 2 diabetes, has been implicated in the activation of the NLR family pyrin domain containing 3 (NLRP3) inflammasome, thereby promoting insulin resistance and systemic inflammation [6,7]. Notably, a strong correlation exists between obesity and the severity of IVD degeneration [8]. One proposed mechanism involves obesity-induced hyperglycemia, which increases reactive oxygen species production and triggers apoptosis in cartilaginous endplate cells [9]. Moreover, obesity is associated with elevated circulating leptin levels [10]. Our previous work demonstrated a positive association between IVD degeneration severity and the expression of leptin and matrix metalloproteinase-1 (MMP-1) in human disc tissues [11]. More recently, we confirmed that leptin induces MMP-1 expression in human cartilage endplate-derived stem cells [12], suggesting that leptin may promote extracellular matrix degradation in IVD via MMP-1 upregulation. Consequently, targeting MMP-1 expression represents a potential strategy for preventing or attenuating IVD degeneration.

Traditional Chinese herbal medicine, a rich source of bioactive compounds, has attracted growing interest for its potential in managing degenerative diseases. Various herbal preparations have demonstrated protective effects on IVD degeneration through anti-inflammatory, antioxidant, and autophagy-enhancing mechanisms [13,14]. *Cinnamomum osmophloeum* Kaneh, a native broadleaf tree species in Taiwan, produces leaf essential oils in which cinnamaldehyde (CA) is the predominant active compound, accounting for approximately 76% of the total oil content [15]. CA has been widely studied for its diverse pharmacological properties. Notably, CA exhibits strong anti-inflammatory and antioxidant activities, including the suppression of lipopolysaccharide-induced reactive oxygen species production and cytokine release in macrophages [16]. It has also been shown to ameliorate dextran sulfate sodium-induced colitis [17] and to inhibit activation of the NLRP3 inflammasome in macrophages infected with *Shigella sonnei* or *Neisseria gonorrhoeae* [18,19]. In metabolic studies, CA promotes lipolysis, helps maintain a balanced leptin/ghrelin ratio, and reduces inflammation in high-fat diet-fed mice [20], while also improving metabolic dysfunction in diabetic models [21]. Furthermore, CA alone or in combination with deferoxamine has been reported to improve outcomes in murine models of intracerebral hemorrhage by reducing inflammation, oxidative stress, and ferroptosis [22]. Additional studies have shown that CA alleviates pulmonary arterial hypertension by mitigating lung injury and fibrosis [23] and attenuates acute myocardial infarction by inhibiting ferroptosis [24]. Moreover, both in silico and experimental evidence support the potential of CA as an anti-cancer agent, particularly in targeting metastasis and invasion through cathepsin B inhibition [25].

In this study, we investigated the effect of CA on MMP-1 expression in leptin-activated human cartilage endplate-derived stem cells and elucidated the molecular mechanisms underlying CA-mediated inhibition of leptin-induced MMP-1 expression. Collectively, our findings highlight the anti-inflammatory and metabolic regulatory potential of CA, supporting its possible application in the prevention or mitigation of IVD degeneration.

## 2. Results

### 2.1. Effect of CA on Leptin-Induced MMP-1 Expression

**Figure 1:**
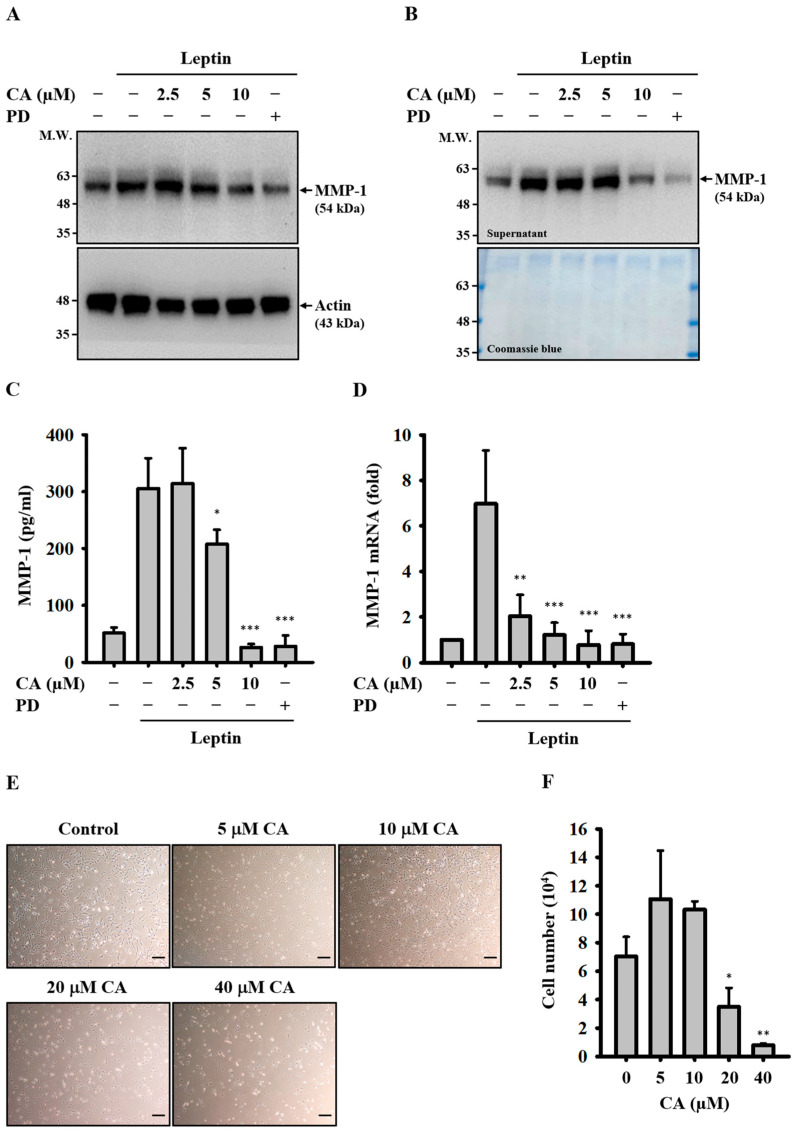
Effect of CA on Leptin-Induced MMP-1 Expression. (**A**–**C**) SV40 cells were pretreated with CA or PD (20 μM) for 30 min, followed by stimulation with leptin for 24 h. Intracellular MMP-1 protein levels were determined by means of Western blot (**A**). Extracellular MMP-1 protein levels were measured by means of Western blot (**B**) and ELISA (**C**). (**D**) SV40 cells were pretreated with CA or PD (20 μM) for 30 min, followed by leptin stimulation for 2 h. MMP-1 mRNA expression was quantified by means of RT-qPCR. (**E**,**F**) SV40 cells were treated with CA for 24 h. Cell morphology was examined by means of light microscopy. Bar = 100 μm (**E**), and cell number was determined using trypan blue exclusion and manual counting (**F**). Representative images of Western blots and cell morphology are shown. Quantitative data from ELISA, RT-qPCR, and cell counts are presented as the mean ± SD from three independent experiments (n = 3). Statistical significance was assessed relative to leptin-stimulated or control groups: * *p* < 0.05, ** *p* < 0.01, and *** *p* < 0.001.

To determine whether CA inhibits leptin-induced MMP-1 expression, SV40 cells were pretreated with CA or MEK inhibitor PD (positive control) for 30 min prior to stimulation with leptin for 24 h. CA reduced both intracellular (Figure 1A) and extracellular (Figure 1B) MMP-1 protein levels in a dose-dependent manner, as determined by means of Western blot. The inhibitory effect of CA on extracellular MMP-1 expression was further confirmed by means of ELISA (Figure 1C). In addition, RT-qPCR analysis revealed that CA suppressed MMP-1 mRNA expression at the transcriptional level (Figure 1D). To evaluate potential cytotoxicity, cell growth was assessed following CA treatment. Concentrations up to 10 µM had no significant effect on cell growth, whereas concentrations ≥20 µM were associated with reduced growth (Figure 1E,F). Collectively, these results indicate that CA significantly inhibits MMP-1 expression in leptin-activated SV40 cells while remaining non-cytotoxic at lower concentrations.

### 2.2. Effect of CA on Leptin-Induced Activation of RhoA and ERK1/2

**Figure 2:**
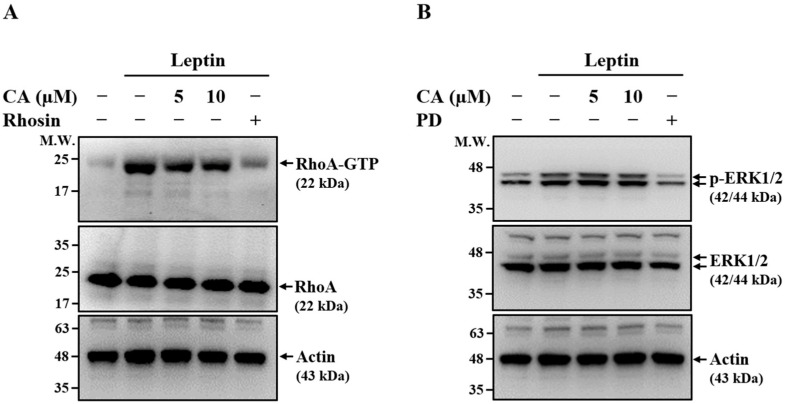
Effect of CA on Leptin-Induced Activation of RhoA and ERK1/2. SV40 cells were pretreated with CA, Rhosin (50 μM), or PD (20 μM) for 30 min, followed by stimulation with leptin for 30 min. (**A**) GTP-bound RhoA levels were measured by means of pull-down assay and detected by means of Western blot. (**B**) ERK1/2 phosphorylation levels were determined by means of Western blot. Representative Western blot images are shown.

To investigate the molecular mechanisms underlying the inhibitory effect of CA on leptin-induced MMP-1 expression, we examined its impact on RhoA and ERK1/2 signaling pathways, both of which have been implicated in regulating MMP-1 expression in leptin-stimulated SV40 cells [12]. SV40 cells were pretreated with CA, the RhoA inhibitor Rhosin, or the MEK inhibitor PD for 30 min, followed by leptin stimulation for 30 min. As shown in Figure 2A, both CA and Rhosin significantly reduced the levels of GTP-bound RhoA (RhoA-GTP) in leptin-stimulated SV40 cells, indicating inhibition of RhoA activation. In contrast, while PD markedly suppressed ERK1/2 phosphorylation, CA had no detectable effect on ERK1/2 phosphorylation (Figure 2B), suggesting that CA does not modulate ERK1/2 activation. Collectively, these results indicate that CA inhibits leptin-induced MMP-1 expression, at least in part, through the suppression of RhoA signaling rather than ERK1/2 pathway modulation.

### 2.3. Effect of CA on Leptin-Induced Activation of NF-κB

**Figure 3:**
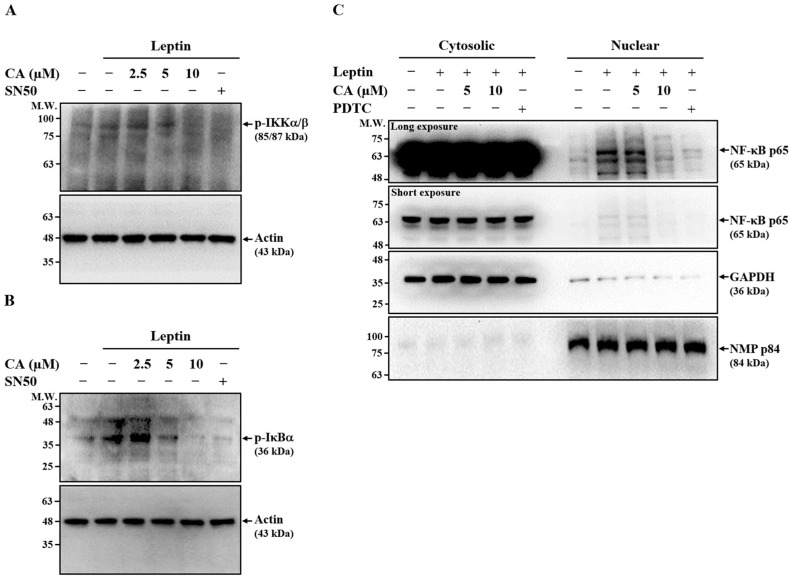
Effect of CA on Leptin-Induced Activation of NF-κB. (**A**,**B**) SV40 cells were pretreated with CA or the NF-κB inhibitor SN50 (10 μM) for 30 min, followed by stimulation with leptin for 30 min. Phosphorylation levels of IKKα/β (**A**) and IκBα (**B**) were determined by means of Western blot. (**C**) SV40 cells were pretreated with CA or the NF-κB inhibitor PDTC (20 μM) for 30 min, followed by leptin stimulation for 2 h. NF-κB p65 levels in cytosolic and nuclear fractions were analyzed by means of Western blot to assess nuclear translocation. Representative Western blot images are shown.

Our previous study demonstrated that NF-κB signaling plays a pivotal role in leptin-induced MMP-1 expression in SV40 cells [12]. To assess whether CA suppresses MMP-1 expression by inhibiting NF-κB activation, SV40 cells were pretreated with CA or the NF-κB inhibitor SN50 (positive control) for 30 min, followed by leptin stimulation for 30 min. As shown in Figure 3A,B, both CA and SN50 markedly reduced the phosphorylation of IKKα/β and IκBα, indicating inhibition of leptin-induced NF-κB activation. To further confirm this effect, we evaluated NF-κB nuclear translocation. SV40 cells were pretreated with CA or the NF-κB inhibitor PDTC for 30 min, followed by leptin stimulation for 2 h. Leptin-induced nuclear translocation of NF-κB p65 was significantly suppressed by both CA and PDTC (Figure 3C). Collectively, these results suggest that CA inhibits leptin-induced MMP-1 expression, at least in part, through downregulation of NF-κB signaling.

### 2.4. Effect of CA on Leptin-Induced Phosphorylation of Leptin Receptor

**Figure 4:**
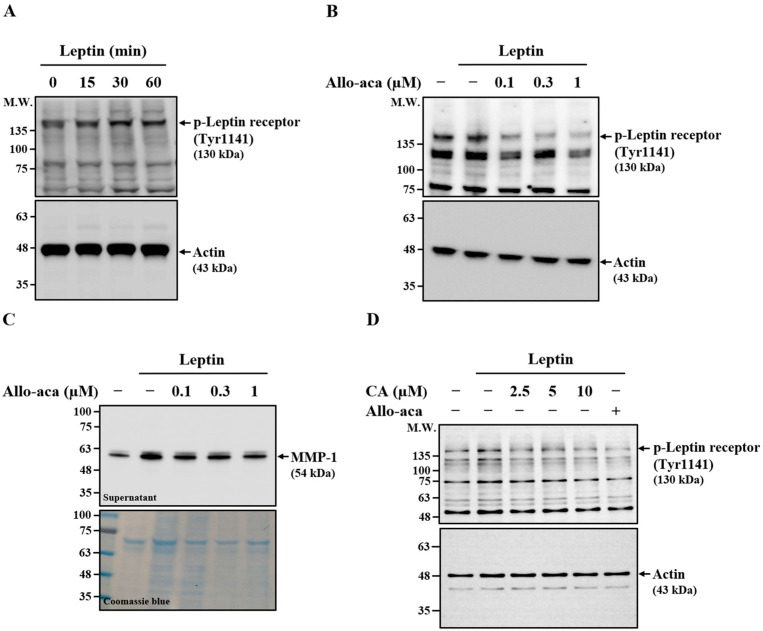
Effect of CA on Leptin-Induced Activation of Leptin Receptor. (**A**) SV40 cells were treated with leptin for 15–60 min, and leptin receptor phosphorylation levels were assessed by means of Western blot. (**B**) SV40 cells were pretreated with the leptin receptor antagonist Allo-aca for 30 min, followed by leptin stimulation for 30 min. Leptin receptor phosphorylation was analyzed by means of Western blot. (**C**) SV40 cells were pretreated with Allo-aca for 30 min, then stimulated with leptin for 24 h. Extracellular MMP-1 protein levels were evaluated by means of Western blot. (**D**) SV40 cells were pretreated with CA or Allo-aca (1 μM) for 30 min, followed by leptin stimulation for 30 min. Leptin receptor phosphorylation was assessed by means of Western blot. Representative Western blot images are shown.

In addition to RhoA, ERK1/2, and NF-κB, we examined whether the leptin receptor contributes to leptin-induced MMP-1 expression in SV40 cells. Leptin stimulation for 30 min increased leptin receptor phosphorylation, which was sustained for up to 60 min, confirming receptor activation (Figure 4A). Treatment with the leptin receptor antagonist Allo-aca significantly reduced receptor phosphorylation in leptin-stimulated cells (Figure 4B), validating its inhibitory effect on receptor activation. To evaluate the role of the leptin receptor in MMP-1 induction, SV40 cells were pretreated with Allo-aca for 30 min prior to leptin stimulation for 24 h. Allo-aca suppressed extracellular MMP-1 expression in a dose-dependent manner (Figure 4C), indicating that receptor activation is required for leptin-induced MMP-1 expression. To determine whether CA inhibits MMP-1 expression by modulating leptin receptor activation, SV40 cells were pretreated with CA or Allo-aca for 30 min before leptin stimulation. Both CA and Allo-aca significantly reduced leptin receptor phosphorylation (Figure 4D).

### 2.5. Effect of CA on Leptin-Induced Activation of JAK2

**Figure 5:**
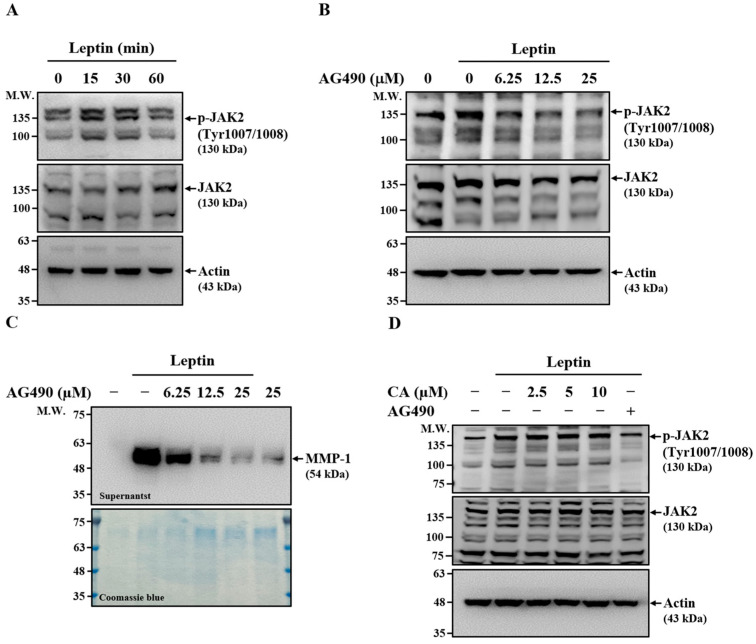
Effect of CA on Leptin-Induced Activation of JAK2. (**A**) SV40 cells were treated with leptin for 15–60 min, and JAK2 phosphorylation levels were analyzed by means of Western blot. (**B**) SV40 cells were pretreated with the JAK2 inhibitor AG490 for 30 min, followed by leptin stimulation for 15 min. JAK2 phosphorylation was assessed by means of Western blot. (**C**) SV40 cells were pretreated with AG490 for 30 min and then stimulated with leptin for 24 h. Extracellular MMP-1 protein levels were measured by means of Western blot. (**D**) SV40 cells were pretreated with CA or AG490 (25 μM) for 30 min, followed by leptin stimulation for 15 min. JAK2 phosphorylation was analyzed by means of Western blot. Representative Western blot images are shown.

JAK2 is a key signaling molecule associated with the leptin receptor and is essential for mediating downstream responses in leptin-stimulated cells [26]. To investigate whether JAK2 is involved in leptin-induced MMP-1 expression, we first examined its activation in SV40 cells. Leptin stimulation increased JAK2 phosphorylation at 15 and 30 min, returning to baseline by 60 min, indicating transient activation (Figure 5A). To further confirm its role, SV40 cells were pretreated with the JAK2 inhibitor AG490 for 30 min before leptin stimulation. AG490 markedly reduced leptin-induced JAK2 phosphorylation (Figure 5B) and dose-dependently suppressed MMP-1 expression in leptin-activated SV40 cells (Figure 5C), supporting a role for JAK2 in MMP-1 upregulation. To determine whether CA inhibits MMP-1 expression through modulation of JAK2 activation, SV40 cells were treated with CA or AG490 for 30 min before leptin stimulation. While AG490 significantly reduced JAK2 phosphorylation, CA had no detectable effect (Figure 5D). These findings indicate that although JAK2 contributes to leptin-induced MMP-1 expression, the inhibitory effect of CA is not mediated through suppression of JAK2 activation.

### 2.6. Effect of CA on Leptin-Induced Activation of STAT3

**Figure 6:**
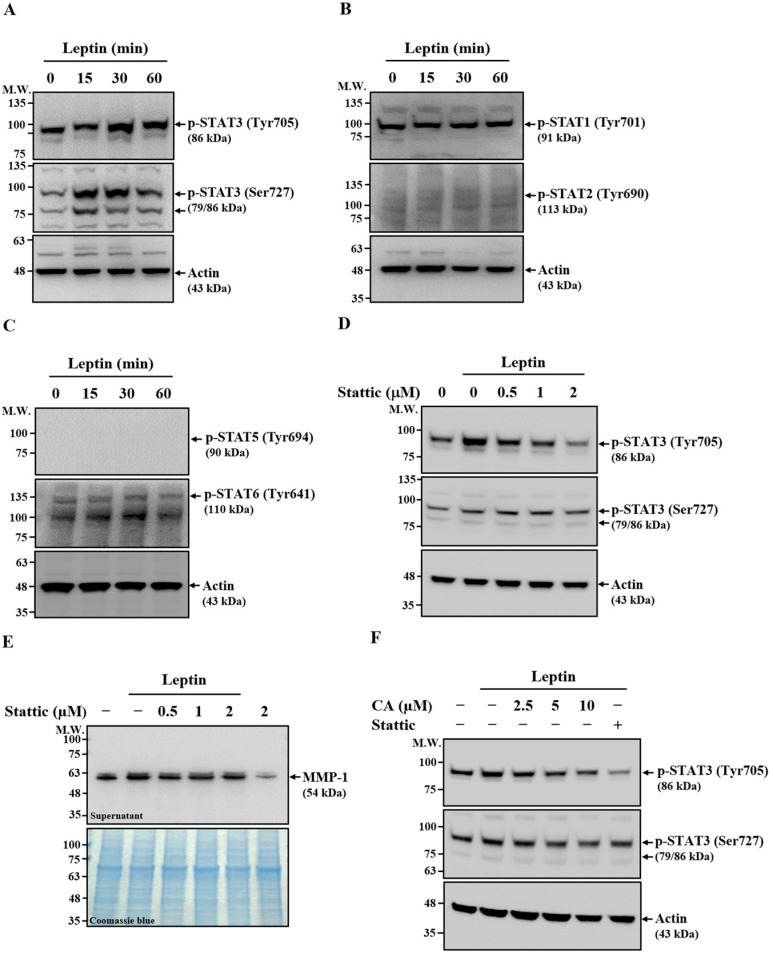
Effect of CA on Leptin-Induced Activation of STAT3. (**A**–**C**) SV40 cells were treated with leptin for 15–60 min, and phosphorylation levels of STAT3 (**A**), STAT1/2 (**B**), and STAT5/6 (**C**) were analyzed by means of Western blot. (**D**) SV40 cells were pretreated with the STAT3 inhibitor Stattic for 30 min, followed by leptin stimulation for 30 min. STAT3 phosphorylation was assessed by means of Western blot. (**E**) SV40 cells were pretreated with Stattic for 30 min and then stimulated with leptin for 24 h. Extracellular MMP-1 protein levels were measured by means of Western blot. (**F**) SV40 cells were pretreated with CA or Stattic (2 μM) for 30 min, followed by leptin stimulation for 30 min. STAT3 phosphorylation was analyzed by means of Western blot. Representative Western blot images are shown.

STAT proteins are critical downstream effectors of the JAK signaling pathway [27]. To assess their potential involvement in leptin-induced MMP-1 expression, SV40 cells were stimulated with leptin for 15–60 min. Western blot analysis showed that leptin increased STAT3 phosphorylation at Tyr705 and Ser727 after 30 and 60 min of stimulation (Figure 6A). In contrast, phosphorylation levels of STAT1 (Tyr701) and STAT2 (Tyr690) (Figure 6B), as well as STAT5 (Tyr694) and STAT6 (Tyr641) (Figure 6C), remained unchanged. These results indicate that leptin selectively activates STAT3 in SV40 cells. To determine the functional role of STAT3 in leptin-induced MMP-1 expression, SV40 cells were pretreated with the STAT3 inhibitor Stattic for 30 min prior to leptin stimulation. Stattic markedly reduced STAT3 phosphorylation at Tyr705 and modestly decreased phosphorylation at Ser727 (Figure 6D). Moreover, Stattic treatment significantly suppressed leptin-induced extracellular MMP-1 expression (Figure 6E), indicating that STAT3 activation is required for MMP-1 induction. Notably, treatment with CA also reduced leptin-induced STAT3 phosphorylation at both Tyr705 and Ser727 (Figure 6F), suggesting that CA inhibits MMP-1 expression in leptin-stimulated SV40 cells, at least in part, through the suppression of STAT3 activation.

### 2.7. Relationship Between JAK2, RhoA, and STAT3 in Leptin-Activated SV40 Cells

**Figure 7:**
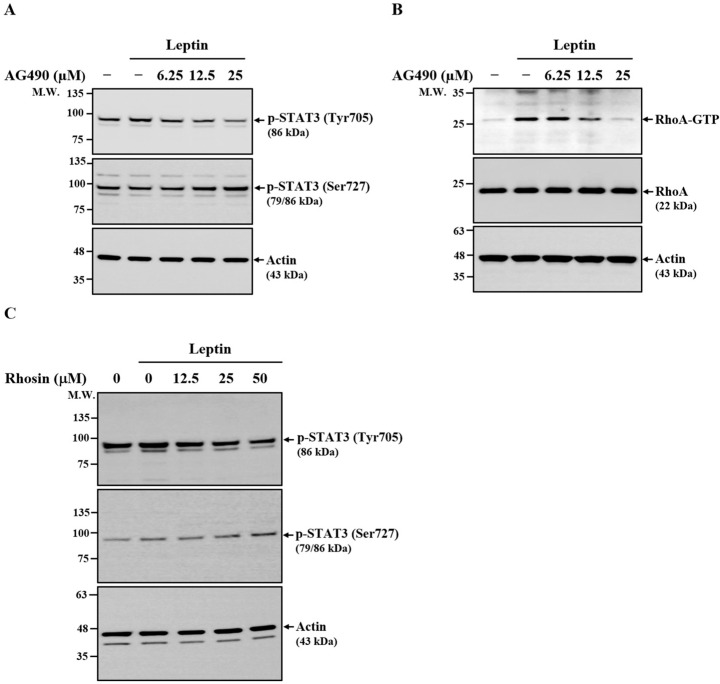
Relationship between JAK2, RhoA, and STAT3 in Leptin-Activated SV40 Cells. (**A**,**B**) SV40 cells were pretreated with AG490 for 30 min, followed by leptin stimulation for 30 min. STAT3 phosphorylation was assessed by means of Western blot (**A**). GTP-bound RhoA levels were measured by means of pull-down assay and detected by means of Western blot (**B**). (**C**) SV40 cells were pretreated with Rhosin for 30 min and then stimulated with leptin for 30 min. STAT3 phosphorylation was assessed by means of Western blot. Representative Western blot images are shown.

To further delineate the signaling pathways regulating leptin-mediated MMP-1 expression in SV40 cells, we examined the relationships among JAK2, STAT3, and RhoA. Treatment with the JAK2 inhibitor AG490 decreased leptin-induced STAT3 phosphorylation at Tyr705, while paradoxically enhancing phosphorylation at Ser727 (Figure 7A), indicating that STAT3 is a downstream effector of JAK2. In addition, AG490 reduced the levels of GTP-bound RhoA in leptin-stimulated cells (Figure 7B), suggesting that RhoA also functions downstream of JAK2. Consistently, inhibition of RhoA with Rhosin decreased STAT3 phosphorylation at Tyr705 while increasing phosphorylation at Ser727 (Figure 7C). Together, these findings demonstrate that both JAK2 and RhoA contribute to the regulation of STAT3 activation in leptin-stimulated SV40 cells.

## 3. Discussion

IVD degeneration is a progressive, age-related process characterized by the depletion of proteoglycans in the nucleus pulposus, resulting in reduced water content and impaired biomechanical function of the disc [1]. Although aging remains the most significant contributor, metabolic imbalance, particularly obesity and diabetes, has emerged as an important risk factor for accelerating disc degeneration [5,8]. Increasing evidence links metabolic disorders with chronic inflammation and catabolic signaling within the disc microenvironment, thereby promoting extracellular matrix degradation [28,29]. MMP-1, a member of the collagenase subfamily, plays a pivotal role in extracellular matrix degradation by cleaving collagens and aggrecan, both of which are crucial for maintaining disc integrity [30]. Elevated expression of MMP-1 has been consistently associated with IVD degeneration [11]. Our previous work demonstrated that leptin, a 16-kDa non-glycosylated adipokine secreted primarily by adipose tissue, is upregulated in obesity [31] and contributes to disc degeneration by inducing MMP-1 expression in human cartilage endplate-derived stem cells [12]. These findings underscore a pathogenic role for leptin-mediated MMP-1 induction in obesity-associated IVD degeneration. In the present study, we provide novel evidence that CA, a bioactive compound derived from *Cinnamomum osmophloeum* [15], significantly inhibits leptin-induced MMP-1 expression in SV40 cells. This observation suggests that CA may serve as a potential therapeutic candidate for mitigating disc degeneration associated with obesity and metabolic disorders. The inhibitory effect of CA on MMP-1 expression further highlights its anti-inflammatory and metabolic regulatory properties, which have been reported in other pathological contexts, including insulin resistance [32] and oxidative stress [33]. Taken together, our findings not only reinforce the role of leptin in mediating obesity-associated disc degeneration through MMP-1 upregulation but also identify CA as a promising natural compound capable of attenuating this pathological process.

Chronic inflammation plays a critical role in the pathogenesis of IVD degeneration [34]. Among the key mediators of inflammation, the NLRP3 inflammasome—a multiprotein complex composed of NLRP3, ASC, and caspase-1—has emerged as a major contributor to inflammatory responses through the activation of caspase-1 and subsequent maturation and secretion of IL-1β. Activation of the NLRP3 inflammasome has been closely linked to the development and progression of various inflammatory diseases, including IVD degeneration [35]. Recent studies have shown that expression levels of NLRP3 inflammasome-related proteins, including NLRP3, caspase-1, and IL-1β, are significantly elevated in the nucleus pulposus tissues of patients with IVD degeneration, and these levels positively correlate with disease severity [36]. Elevated IL-1β expression contributes to disc degeneration by inhibiting the synthesis and promoting the degradation of the extracellular matrix, ultimately leading to structural deterioration of the disc [37]. Furthermore, caspase-1-mediated pyroptosis in nucleus pulposus cells has been implicated in the exacerbation of IVD degeneration pathology [38], reinforcing the role of the NLRP3 inflammasome as a key pathogenic mechanism in IVD degeneration [39]. Given the central role of NLRP3 inflammasome activation in IVD degeneration, therapeutic strategies targeting this pathway have gained increasing attention. Natural compounds with anti-inflammatory properties have shown promise in modulating NLRP3 activity and may represent novel therapeutic agents for IVD degeneration treatment [40,41]. In our recent studies, we demonstrated that CA alleviates dextran sulfate sodium-induced colitis in mice and reduces inflammatory responses in *Shigella sonnei*- and *Neisseria gonorrhoeae*-infected macrophages by inhibiting NLRP3 inflammasome activation [17,18,19]. These findings suggest that CA may have therapeutic potential in IVD degeneration, not only by suppressing leptin-induced MMP-1 expression, but also by inhibiting NLRP3 inflammasome activation.

The leptin receptor lacks intrinsic kinase activity and relies on receptor-associated JAK2 for phosphorylation of specific tyrosine residues within its intracellular domain [42]. In our study, CA inhibited leptin-induced leptin receptor phosphorylation without affecting leptin-induced JAK2 phosphorylation. This suggests that CA does not interfere with JAK2 activation per se, but rather modulates the phosphorylation of the leptin receptor itself. One plausible explanation is that CA may hinder the accessibility or conformational orientation of specific tyrosine residues on the leptin receptor that serve as phosphorylation sites for JAK2. Such an effect could occur through direct interaction with the receptor or by altering the local receptor microenvironment, thereby preventing efficient transfer of phosphate groups from activated JAK2 to the receptor’s intracellular tail. This mechanism differs from that of Allo-aca, a potent leptin receptor antagonist peptide, which inhibits leptin-induced receptor phosphorylation by competitively blocking leptin binding to the receptor [43]. Since CA did not reduce JAK2 phosphorylation in our experiments, it is unlikely to block ligand–receptor interaction at the extracellular domain; instead, it may selectively impair the phosphorylation step at the intracellular interface between the receptor and JAK2. Further studies, such as site-specific mutagenesis of receptor tyrosine residues, structural modeling of CA–receptor interactions, or biophysical analyses of receptor conformation, would be valuable for clarifying whether CA acts directly on the receptor’s intracellular phosphorylation sites or indirectly through membrane-associated regulatory proteins.

**Figure 8:**
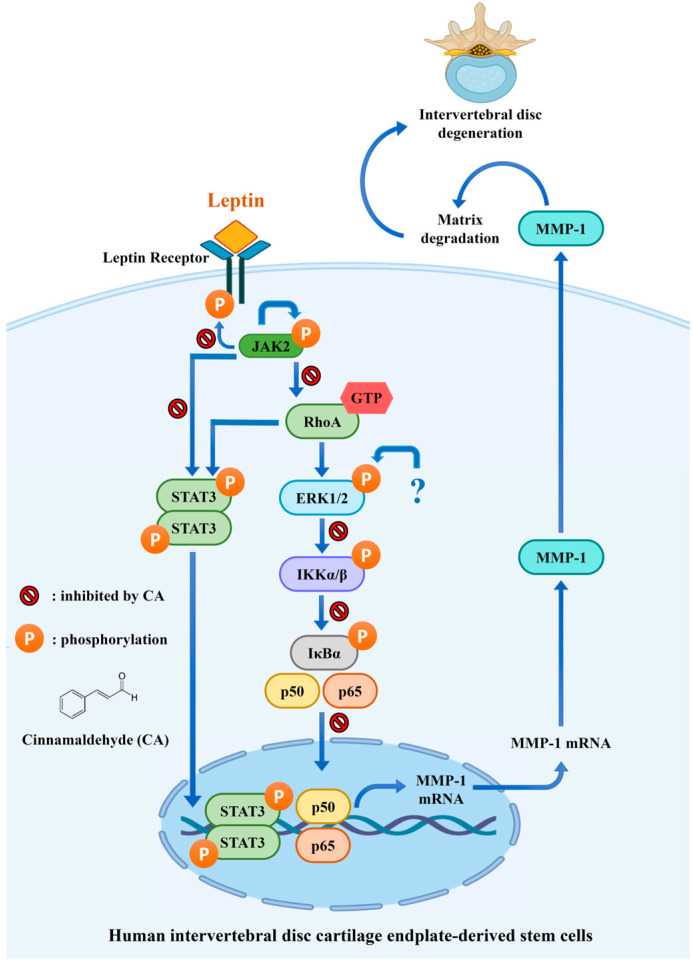
An overview of the proposed and established mechanisms by which CA inhibits MMP-1 expression in SV40 cells. Proteins represented with a blue question mark are currently unidentified.

In our previous study, we demonstrated that leptin activates both RhoA and ERK1/2 in SV40 cells, with RhoA functioning as an upstream regulator of ERK1/2 [12]. Based on this signaling hierarchy, we anticipated that inhibition of RhoA activation by CA would also lead to reduced ERK1/2 phosphorylation. However, in the present study, CA significantly suppressed leptin-induced RhoA activation but had no detectable effect on ERK1/2 phosphorylation. This discrepancy suggests that ERK1/2 activation in leptin-stimulated SV40 cells may not depend solely on RhoA signaling. ERK1/2 can be phosphorylated and activated through several alternative pathways. For example, leptin can activate ERK1/2 via the SHP2/Grb2/SOS/MEK cascade [44]. In addition, cross-talk with other receptor systems, such as leptin-mediated epidermal growth factor receptor transactivation, has been shown to sustain ERK1/2 activity independently of RhoA [45]. It is therefore possible that CA selectively inhibits the RhoA/NF-κB pathway, which is more directly linked to MMP-1 expression, while leaving the JAK2/SHP2/ERK1/2 axis or other compensatory mechanisms intact. Moreover, distinct subcellular pools of RhoA may regulate cytoskeletal or transcriptional processes without necessarily converging on ERK1/2 phosphorylation, which could also explain the differential effects of CA. Taken together, these findings indicate that while RhoA contributes to leptin-induced MMP-1 expression, ERK1/2 activation in this context may be maintained through RhoA-independent mechanisms. Future studies using specific inhibitors of SHP2, Ras, or EGFR transactivation, as well as temporal analyses of pathway activation, will be important for delineating the precise signaling networks by which leptin regulates ERK1/2 and for clarifying how CA selectively interferes with leptin signaling. An overview of the proposed and established mechanisms by which CA inhibits MMP-1 expression in SV40 cells is provided in Figure 8.

CA is the predominant active compound found in the leaves of *Cinnamomum osmophloeum* Kaneh, a food-grade material approved by the Ministry of Health and Welfare, Taiwan. Although CA has a long history of use as a food additive, its safety profile has also been supported by toxicological studies. In a previous study, oral administration of CA at a dose of 480 mg/(kg·day) for 42 days in mice resulted in a 100% survival rate, with no significant histopathological changes observed in the kidney, lung, or liver [46]. However, a major limitation of the present study is the lack of in vivo data evaluating the therapeutic efficacy of CA in animal models of IVD degeneration. Future investigations should aim to elucidate the detailed molecular mechanisms through which CA modulates leptin signaling pathways and to assess its potential therapeutic effects in well-established in vivo models of IVD degeneration.

## 4. Materials and Methods

### 4.1. Reagents

The trans-Cinnamaldehyde (CAS number: 14371-10-9; IUPAC name: (2E)-3-Phenylprop-2-enal; ≥99%), pyrrolidine dithiocarbamate (PDTC), AG490, Stattic, Rhosin, SN50, and antibody against phospho-Janus Kinase 2 (JAK2) (Tyr1007/1008) were purchased from Sigma-Aldrich (St. Louis, MO, USA). Recombinant human leptin protein (398-LP) was obtained from R&D Systems (Minneapolis, MN, USA). The enzyme-linked immunosorbent assay (ELISA) kit for human MMP-1, enhanced chemiluminescence substrates, and antibody against phospho-Leptin Receptor (Tyr1141) were procured from Thermo Fisher Scientific (Waltham, MA, USA). The Coomassie stain kit (iBlue, Shanghai, China) was purchased from GeneDireX (Taoyuan, Taiwan). We acquired the antibody against human MMP-1 from GeneTex (Irvine, CA, USA). The antibody against ERK1/2 was sourced from Taiclone (Taipei, Taiwan). Antibodies against phospho-ERK1/2, phospho-IKKα/β, phospho-IκBα, NF-κB p65, GAPDH, JAK2, phospho- signal transducer and activator of transcription (STAT) 3 (Tyr705), phospho-STAT3 (Ser727), phospho-STAT1 (Tyr701), phospho-STAT2 (Tyr690), phospho-STAT5 (Tyr694), phospho-STAT6 (Tyr641), and PD98059 (PD) were obtained from Cell Signaling Technology (Danvers, MA, USA). We acquired Nuclear Matrix Protein p84 (NMP p84) from ABclonal (Boston, MA, USA). Prigrow IV medium (TM004) and fetal bovine serum were purchased from Applied Biological Materials (Richmond, BC, Canada). Rho Assay Reagent (Rhotekin RBD, agarose) and Amicon Ultra-4 centrifugal filter unit (UFC801024) were procured from Merck (Taipei, Taiwan). Allo-aca was purchased from MedChemExpress (Monmouth Junction, NJ, USA). The Nuclear/Cytosol Fractionation Kit was purchased from BioVision (Milpitas, CA, USA).

### 4.2. Cell Culture

The human intervertebral disc cartilage endplate-derived stem cell line (SV40 cells) was obtained from Applied Biological Materials (Richmond, BC, Canada). SV40 cells were grown and subcultured every 2 to 3 days in Prigrow IV medium, which was enriched with 10% heat-inactivated fetal bovine serum, and kept at 37 °C in a 5% CO_2_ incubator.

### 4.3. Effect of CA on Leptin-Induced MMP-1 Expression

For the assessment of MMP-1 protein expression, SV40 cells were pretreated with CA (2.5–10 μM), MEK inhibitor PD (20 μM), or vehicle control (0.1% DMSO) for 30 min, followed by stimulation with leptin (100 ng/mL) or vehicle control (0.1% PBS) for 24 h. Intracellular MMP-1 protein levels were determined by means of Western blot analysis of cell lysates. Extracellular MMP-1 protein levels were evaluated by detecting MMP-1 in culture supernatants using either Western blot (after concentration with an Amicon Ultra-4 centrifugal filter unit) or ELISA. For MMP-1 mRNA expression analysis, SV40 cells were treated with CA (2.5–10 μM), PD (20 μM), or vehicle control for 30 min, followed by stimulation with leptin (100 ng/mL) or vehicle control for 2 h. Total RNA was extracted, reverse transcribed (RT), and MMP-1 mRNA expression was quantified by means of quantitative real-time polymerase chain reaction (qPCR). MMP-1 mRNA expression data were presented as relative expression levels normalized to those of glyceraldehyde-3-phosphate dehydrogenase (GAPDH). The following primers were employed: MMP-1, forward: 5′-ATGCTTTTCAACCAGGCCCA-3′; MMP-1, reverse: 5′-AGTCCAAGAG AATGGCCGAG-3′; GAPDH, forward: 5′-TTCCAGGAGCGAGATCCCT-3′; GAPDH, reverse: 5′-CACCCATGA CGAACATGGG-3′.

### 4.4. Effect of CA on Leptin-Induced Activation of RhoA

SV40 cells were pretreated with CA (5–10 μM), RhoA inhibitor Rhosin (50 μM), or vehicle control for 30 min, followed by stimulation with leptin (100 ng/mL) or vehicle control for an additional 30 min. RhoA activation was assessed by quantifying the level of GTP-bound RhoA using the Rho Assay Reagent (Rhotekin RBD, agarose), as previously described [12].

### 4.5. Effect of CA on Leptin-Induced Phosphorylation of ERK1/2

SV40 cells were pretreated with CA (5–10 μM), PD (20 μM), or vehicle control for 30 min, followed by stimulation with leptin (100 ng/mL) or vehicle control for 15 min. Phosphorylation levels of ERK1/2 were determined by means of Western blot analysis.

### 4.6. Effect of CA on Leptin-Induced Activation of NF-κB

To assess the phosphorylation of IKKα/β and IκBα, SV40 cells were pretreated with CA (2.5–10 μM), SN50 (10 μM), or vehicle control for 30 min, followed by stimulation with leptin (100 ng/mL) or vehicle control for an additional 30 min. Phosphorylation levels of IKKα/β and IκBα were determined by means of Western blot analysis. To evaluate NF-κB p65 nuclear translocation, SV40 cells were pretreated with CA (5–10 μM), PDTC (20 μM), or vehicle control for 30 min, followed by stimulation with leptin (100 ng/mL) or vehicle control for 2 h. Cytoplasmic and nuclear fractions were prepared using the Nuclear and Cytoplasmic Extraction Kit according to the manufacturer’s instructions [12]. NF-κB p65 levels in nuclear and cytosolic fractions were analyzed by means of Western blot. Fraction purity was confirmed by detecting the nuclear marker NMP p84 and the cytosolic marker GAPDH.

### 4.7. Effect of CA on Leptin-Induced Phosphorylation of Leptin Receptor, JAK2, and STATs

To examine leptin-induced phosphorylation of the leptin receptor, JAK2, and STATs, SV40 cells were stimulated with leptin (100 ng/mL) for 15–60 min or treated with vehicle control for 60 min. Phosphorylation levels of the leptin receptor, JAK2, and STATs were determined by means of Western blot analysis. To assess the effect of CA on leptin-induced protein phosphorylation, SV40 cells were pretreated with CA (2.5–10 μM), leptin receptor antagonist peptide Allo-aca (0.1–1 μM), JAK2 inhibitor AG490 (6.25–25 μM), STAT3 inhibitor Stattic (0.5–2 μM), or vehicle control for 30 min, followed by stimulation with leptin (100 ng/mL) or vehicle control for an additional 15 min (for JAK2) or 30 min (for leptin receptor and STATs). Phosphorylation levels were then evaluated by means of Western blot analysis.

### 4.8. Relationship Between JAK2, RhoA and STAT3 in Leptin-Activated SV40 Cells

To investigate the roles of JAK2 and RhoA in leptin-induced STAT3 activation, SV40 cells were pretreated with AG490 (6.25–25 μM), Rhosin (12–50 μM), or vehicle control for 30 min. Cells were then stimulated with leptin (100 ng/mL) or vehicle control for an additional 30 min. STAT3 phosphorylation levels were assessed by means of Western blot. The levels of active, GTP-bound RhoA were measured using a RhoA activation assay kit, according to the manufacturer’s protocol, as previously described [12].

### 4.9. Statistical Analysis

Graphs were generated using GraphPad Prism version 6.01, and statistical analyses were performed using IBM SPSS Statistics version 24. One-way analysis of variance (ANOVA) was applied for comparisons among multiple groups. Data are presented as the mean ± standard deviation (SD) from independent experiments. Statistical significance was defined as *p* < 0.05 (* *p* < 0.05; ** *p* < 0.01; *** *p* < 0.001). The “n” values in the figure legends refer to the number of independent cell culture preparations used in the experiments.

## 5. Conclusions

This study demonstrates that CA, a natural compound derived from *Cinnamomum osmophloeum* Kaneh, effectively suppresses leptin-induced MMP-1 expression in human IVD cartilage endplate-derived stem cells. Mechanistically, CA inhibits key components of the leptin signaling cascade, including phosphorylation of the leptin receptor and STAT3, and activation of RhoA and NF-κB, while having no effect on JAK2 and ERK1/2 phosphorylation. These findings suggest that CA modulates specific signaling pathways involved in leptin-mediated extracellular matrix degradation. Given its ability to attenuate molecular events contributing to IVD degeneration, CA represents a promising therapeutic candidate for obesity-associated disc degeneration. Further in vivo studies are warranted to validate its efficacy and safety.

## Data Availability

Data will be made available upon request.

## Abbreviations

The following abbreviations are used in this manuscript:

IVD	Intervertebral disc
MMP-1	Matrix metalloproteinase-1
SV40 cells	Human intervertebral disc cartilage endplate-derived stem cells
RhoA	Ras homolog family member A
JAK2	Janus Kinase 2
STAT	Signal transducer and activator of transcription
CA	Cinnamaldehyde
PD	PD98059
PDTC	Pyrrolidine dithiocarbamate
NLRP3	NLR family pyrin domain containing 3
NMP p84	Nuclear matrix protein p84
RT	Reverse transcribed
qPCR	Quantitative real-time polymerase chain reaction
GAPDH	Glyceraldehyde-3-phosphate dehydrogenase
ANOVA	One-way analysis of variance
SD	Standard deviation
ELISA	Enzyme-linked immunosorbent assay
